# The inhibitory effect of boric acid on hypoxia-regulated tumour-associated carbonic anhydrase IX

**DOI:** 10.1080/14756366.2022.2072837

**Published:** 2022-05-10

**Authors:** Zainab Saad Yusuf, Tugba Kevser Uysal, Ender Simsek, Alessio Nocentini, Sameh Mohamed Osman, Claudiu T. Supuran, Özen Özensoy Güler

**Affiliations:** a Department of Medical Biology, Faculty of Medicine, Ankara Yildirim Beyazit University, Ankara, Turkey; b Neurofarba Department, Section of Pharmaceutical Chemistry, Universita degli Studi di Firenze, Florence, Italy; c Chemistry Department, College of Science, King Saud University, Riyadh, Saudi Arabia

**Keywords:** Boric acid, carbonic anhydrase, isoform CA IX, enzyme inhibitor

## Abstract

Carbonic anhydrases (EC 4.2.1.1) catalyse the reversible hydration of CO_2_ into bicarbonate and protons. As a hypoxia-sensitive and tumour-associated isoform, isoform CA IX, is significantly overexpressed in various malignancies, being a validated target for new anticancer/antimetastatic drugs. A multitude of studies has shown that CA IX inhibition decreases cancer cell proliferation and metastasis through pHe/pHi modulation and enhancement of ferroptosis among others. Numerous studies demonstrated increased efficacy of cytotoxic drugs combined with CA inhibitors (CAIs) in various cancer types. We tested the inhibitory effect of boric acid (BA), an inorganic Lewis acid, on CA IX as well as other isoforms (CA I, II, and XII). BA acted as a millimolar *in vitro* CAI, decreased proliferation of two cancer cell lines, although not strong correlations between the *in vitro* inhibition and *in vivo* effects were observed. The mechanism of antiproliferative action of BA should be investigated in more detail.

## 1. Introduction

Carbonic anhydrases (CAs, EC 4.2.1.1) are a metalloenzyme superfamily that catalyses the reaction between carbon dioxide (CO_2_), bicarbonate (HCO_3_^−^), and protons (H^+^)^1^. Among its isoforms, CA IX is defined as a hypoxia-sensitive tumour-associated protein due to its abundant expression in tumour tissues^2^. CA IX plays a critical role in the pH regulation of the cancer cell microenvironment. It transports HCO_3_^-^ anions into the cytoplasm by catalysing the hydration of CO_2_ thereby creating a slightly alkaline cytoplasm which is involved in ferroptosis as well as an acidic external microenvironment which favours the growth of tumour cells and enhances their metastases capacities^3^. Cancer cells thrive in such an acidic microenvironment, but a too low pH and excessive acidification may also lead to necrosis of these cells^3^. Extensive *in vitro*, cell culture and animal model studies have demonstrated that CA IX is a validated target for drug design of new therapeutic and diagnostic tools in cancer treatment^4^^,^^5^.

Hypoxia has been recognised as a characteristic of tumours caused by defective angiogenesis^6^. At the molecular level, adaptation of tumour cells to the hypoxic stress is largely regulated by the hypoxia-inducible factor (HIF), a transcription factor that accumulates in response to reduced cellular oxygen levels^7^. HIF-1 is responsible for the transcription of target genes that cause cell proliferation, angiogenesis (activates the transcription of vascular endothelial growth factor (VEGF) and its receptor VEGFR1, for promoting endothelial cell proliferation and blood vessel formation), metabolic adaptation (HIF directly up-regulates glucose transporters 1–3 (GLUT-1 − 3) and enzymes of the glycolytic pathway. It also induces pyruvate dehydrogenase kinase 1 (PDK1) expression, and inhibits pyruvate dehydrogenase (PDH), which converts pyruvate to Acetyl-CoA, cell survival, apoptosis resistance (expression of survivin, an apoptosis inhibitor) invasion, and metastasis^6–8^. The expression CA IX is also highly influenced by hypoxia, because another function HIF-1 is to cause the overexpression of CA IX in cancer^8^.

Many studies on potential CA inhibitors (CAIs), such as sulphonamides, sulfamates, sulfamides, carboxylic acids, phenols, polyamines, diols, borols, boronic acids, coumarins, and sulfocoumarins have yielded interesting results for many pharmacological application, among which antiglaucoma, antiepileptoic, antobesity, antitumor, anti-ischemic, and anti-infective effects of such enzyme inhibitors^4^^,^^7^^,^^9^. Studies on antitumor sulphonamides and sulfamates led to interesting results, with a sulphonamide inhibitor (SLC-0111) and some monoclonal antibodies in phase I/II clinical trials^10^. There are currently four known mechanisms of CA inhibition; (i) inhibitors that bind to the metal ion, such as sulphonamides, sulfamates, sulfamides, many inorganic anions, dithio-monothiocarbamates and xanthates, carboxylates and hydroxamates, boronic acids and borols, and diols^9^; (ii) inhibitors anchoring to the non-protein metal ion ligand, i.e. phenols, polyamines, hydrolysed sulfocoumarins (i.e. sulphonic acids), some carboxylates, 2-thioxocoumarins^9^; (iii) compounds that blocking the active site entrance (coumarins and their derivatives)^9^; (iv) inhibitors binding outside the active site (some carboxylates)^9^.

Boric acid (B(OH)_3_, BA) is a weak Lewis acid and highly electrophilic derivative, making it an interesting compound for investigations as enzyme inhibitor^11^. The boron atom has an empty p-orbital that allows it to form complexes with amino and hydroxyl moieties present in various biomolecules, such as enzymes, nucleotides, vitamins, and carbohydrates through electron donor-acceptor (covalent) bonds. This property is retained in many boron(III) compounds. Some enzymes inhibited by B(OH)_3_ and some of its organic derivatives, such as boronic acids, include peptidases, proteases, proteosomases, arginase, nitric oxide synthase, and transpeptidases^12^.

Though no existing studies were reported on B(OH)_3_ as a CAI, especially as a CA-IX inhibitor, there are studies on other boron-containing CAIs, such as diarylborols, phenylboronic acids, benzoxaboroles, bis-benzoxaboroles, bortezomib, sodium tetraborate (borax) and dipotassium-trioxohydroxy tetrafluorotriborate, that showed promising results^12^. The inhibition mechanism for these boron-containing CAI consists in the coordination to the catalytic metal ion from the enzyme active site^12^.

We hypothesise that B(OH)_3_ can also inhibit CAs. Such an inhibition, if detected, might be similar to the above-mentioned boron derivatives (diarylborols, phenylboronic acids, benzoxaboroles, etc.), although being a small molecule without an organic scaffold, we expect BA to behave as a weak CAI. The purpose of this study was to investigate the effect of BA on several CA isoforms, including CA IX, which might lead to an explanation of some of its antiproliferative properties. We examined CA IX levels in BA-treated cancer cell cultures under normoxic and hypoxic conditions. For comparison, HT29 (human colon cancer) and A375 (human malignant melanoma) cell cultures were also treated with acetazolamide (AZA), a sulphonamide CAI mainly used as an antiglaucoma and diuretic drug, which also shows antiproliferative activity in some cancer cell lines^6^.

## 2. Materials and methods

### 2.1. Materials and kits

The cell lines HT29 (human colon cancer) and A375 (human malignant melanoma) were purchased from American Type Cell Collection (ATCC, Washington, DC). We used a hypoxic chamber to create a hypoxic environment for the experiments. The WST-1 reagent for the cytotoxicity test and the CA IX ELISA and a HIF-1α ELISA kits were commercially available (Shanghai Coon Koon Biotech co ltd., Shanghai, China) and were used to determine the levels of CA IX and HIF-1α in HT29 and A375 cultures grown under hypoxic and normoxic conditions.

### 2.2. Cell culture in hypoxic condition

The cell lines used in the experiments, HT29 (ATCC HB-38) and A375 (ATCC CRL 1619), were grown in Dulbecco’s modified Eagle medium (DMEM) supplemented with 10% foetal bovine serum (FBS) and treated with 1% penicillin-streptomycin. Cells were incubated at 37 °C and 5% CO_2_ in 25cm^3^ flasks. On reaching 80–90% confluence, cell harvesting was performed by washing cells in phosphate-buffered saline (PBS) and then detached from the flask surface using trypsin-EDTA. Cell count was determined using a hematocytometer. Two separate cultures each for both HT29 and A375 were grown, one under normoxic conditions and the second one under hypoxic conditions. The normoxic cultures for HT29 and A375 were grown and incubated at normoxic conditions (37 °C and 5% CO_2_). The hypoxic cultures (2% O_2_) of HT29 and A375 were cultured by using a hypoxic chamber with a gas mixture of 10% CO_2_, 88% N_2,_ and 2% O_2_ (Stemcell Technologies, Cambridge, UK) according to the manufacturer’s instruction. The hypoxic chamber was placed in a 37 °C incubator for 4 h. Hypoxia conditions were confirmed by detecting HIF-1α protein level by using an ELISA kit (Shangai Koon, Shanghai, China).

### 2.3. Cytotoxicity assay and inhibitor treatment

WST-1 reagent was used to measure mitochondrial dehydrogenase activity and determine cell viability. A 0.5 M stock of B(OH)_3_ was prepared by dissolving 0.309 g of B(OH)_3_ in 10 ml of distilled water. HT29 and A375 cells were seeded on 96-well plates and incubated for 24 h, allowing them to adhere to the wells before treatment with B(OH)_3_. Cells were then treated with 2.5, 5, 10, 25 50, 100, and 200 mM B(OH)_3_ and topped to 100 µL with a prepared cell medium (one well remaining as an untreated, negative control). The plates were then incubated for 24 and 48 h periods. After each period, 10 ml WST-1 reagent was added to each well and then the plate was incubated for another 3–4 h at 37 °C with 5% CO_2_. The plates were read by using an Epoch microplate reader (BioTek, Cambridge, MA) with a wavelength of 450 nm. The results were analysed by using GraphPad Prism version 9 (Graphpad Software Inc., La Jolla, CA), for determining the IC_50_ values, with AZA as standard CA inhibitor. HT29 and A375 cells were seeded 0.3 × 10^6^ cells/well into two separate six-well plates. On each well-plate, one column was treated with BA, the second was treated with AZA, and the third column was left untreated, serving as a control. The doses used for BA and AZA were the IC_50_ determined after 24 h from the WST assay. The plates were then set aside to incubate for 24 h under normoxic conditions (37 °C and 5% CO_2_).

### 2.4. Determination of CA IX and HIF-1α levels

For determination of protein levels, cell lysates of the six-well plates were seeded one day before the experiment and treated with the determined IC_50_ concentration of BA or AZA for 24 h under normoxic and hypoxic conditions. Cells were washed with PBS and then were treated in a lysis buffer (1% NP-40, 50 mM Tris, and 150 mM NaCl) with protease inhibitors on ice. The lysates were collected by centrifugation at 13,000 × *g* for 15 min at 4 °C. These supernatants were collected, and the aliquots were stored at −20 °C. These supernatants were used to detect the levels of CA IX and HIF-1α levels using commercially available enzyme-linked immunosorbent assay (ELISA) kits. These protein levels were determined by using CA IX ELISA kit and HIF1α ELISA kit according to the manufacturer’s instructions. Of 40 µL of the cell lysates was seeded into the microplates and with 10 µL of protein conjugate and 50 µL of streptavidin-horseradish to reach a volume of 100 µL. The microplates were incubated at 37 °C for 1 h and then washed five times with buffer. Of 50 µL of chromogen A and 50 µL of chromogen B were added to the plates and incubated at 37 °C for 10 min. Of 50 µL of the stop solution was then added and the microplates were read at 450 nm using the Epoch micro-plate reader (BioTek, Cambridge, MA).

### 2.5. Inhibition data of human CA isoforms CA I, II, IX, and XII with boric acid

The standard sulphonamide inhibitor AAZ and BA were tested as hCA I, II, IX, and XII inhibitors by a stopped-flow CO_2_ hydrase assay^13^. Phenol red (at a concentration of 0.2 mM) was used as indicator, working at the absorbance maximum of 557 nm, with 20 mM Hepes (pH 7.5) as buffer and 20 mM Na_2_SO_4_ (for maintaining constant the ionic strength), following the initial rates of the CA-catalysed CO_2_ hydration reaction for a period of 10 − 100 s. The CO_2_ concentrations ranged from 1.7 to 17 mM for the determination of the kinetic parameters and inhibition constants. For each inhibitor, at least six traces of the initial 5 − 10% of the reaction have been used for determining the initial velocity. The uncatalysed rates were determined in the same manner and subtracted from the total observed rates. Stock solutions of BA (or other inhibitors) (100 mM) were prepared in distilled – deionised water, and dilutions up to 0.01 nM were done thereafter with the assay buffer. Inhibitor and enzyme solutions were preincubated together for 6 h at room temperature prior to assay in order to allow for the formation of the E–I complex. The inhibition constants were obtained by nonlinear least-squares methods using PRISM 3 and the Cheng–Prusoff equation, as reported earlier^12^, and represent the mean from at least three different determinations. All CA isoforms were recombinant ones obtained in-house as reported earlier^12^ and their concentrations in the assays system were of the order of 10–12.5 nM.

### 2.6. Statistical analysis

All statistical analysis was performed using Graphpad Prism version 9. All data were expressed as mean ± SD. A comparison between more than two groups was analysed using one-way ANOVA and *post hoc* Tukey test; a two-tailed student’s t-test was used for comparison between two groups. Statistical significance level was accepted as *p* < .05.

## 3. Results

### 3.1. Determination of cell viability

**Figure 1.**
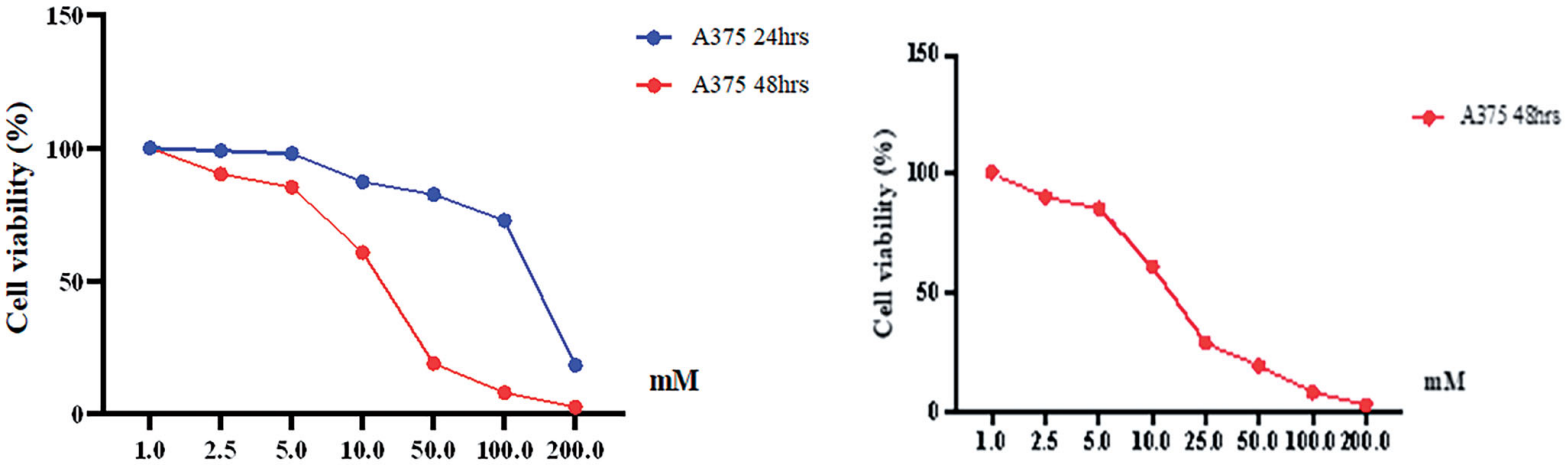
Cell viability percentages of A375 cell lines treated with ascending boric acid concentrations according to the WST-1 assay results.

**Figure 2.**
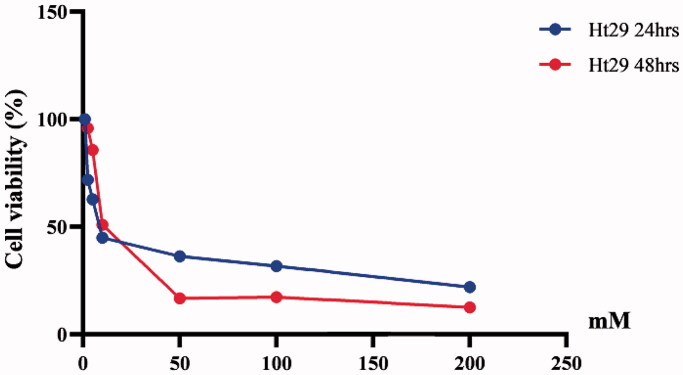
Cell viability percentages of HT29 cell lines treated with ascending boric acid concentrations according to the WST-1 assay results.

A WST-1 assay was carried out to verify cell viability after 24 and 48 h. The results presented in Figures 1 and 2 indicate that BA induced dose-dependent cytotoxicity in both HT29 and A375 cell lines. BA concentration of 2.5, 5, 10, 50, 100, and 200 mM decreased cell viability of A375 and HT29 (Figures 1 and 2). IC_50_ was determined at 128 and 15.5 mM for A375 after 24 and 48 h, respectively. For HT29 IC_50_ was determined to be of 19.6 and 23.0 mM after 24 and 48 h, respectively. IC_50_ doses of BA determined after 24 h, 128 mM for A375 and 23.0 mM for HT29 were then selected and used as inhibitor treatment doses for the cells.

### 3.2. Assessment of hypoxia (HIF-1α) levels

**Figure 3.**
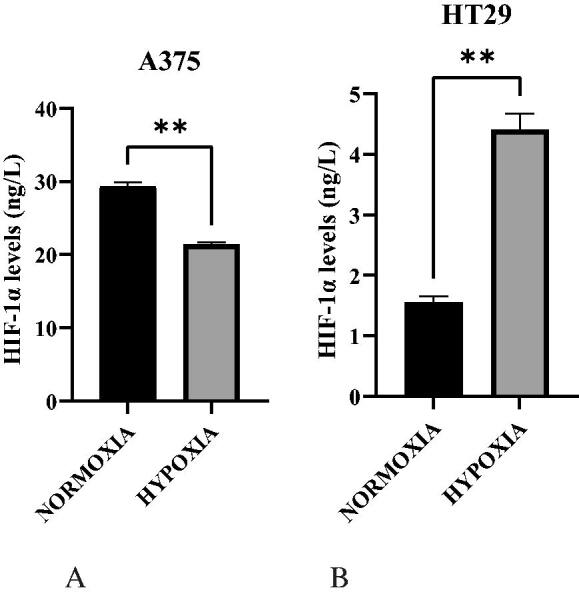
A. HIF-1α levels in normoxic and hypoxic A375 cells. B. HIF-1α levels in normoxic and hypoxic HT29 cells.

While confirming hypoxia using ELISA, the HIF-1α level in normoxic A375 was significantly higher than in hypoxic A375 (Figure 3(A), *p* < .05). This was not completely unexpected as a study in 2010 demonstrated the increase of HIF activity in malignant melanoma under normoxic conditions unlike other tumors^14^. In contrast, the HIF-1α level in hypoxic HT29 was exceedingly higher than in normoxic HT29 (Figure 3(B), *p* < .05) this was also expected, it is the results seen in most solid tumours.

### 3.3. Determination of CA IX levels

**Figure 4.**
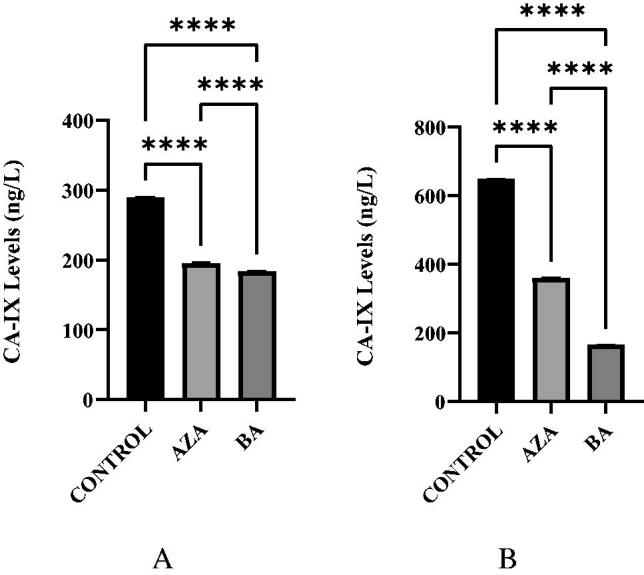
A. CA IX levels in AZA, BA, and Control A375 hypoxic cell cultures. CA IX levels in AZA and BA treated cells were significantly reduced when compared to Control (*p* < .0001). B. CA IX levels in AZA, BA, and control A375 normoxic cell cultures. CA IX levels are significantly reduced in AZA-treated cells and more so in BA-treated cells compared to Control (*p* < .0001).

CA IX enzyme level was significantly decreased after treatment with AZA and BA under hypoxic conditions in A375 cell cultures (Figure 4(A)). Both BA and AZA showed a positive correlation for the inhibition of CA IX in A375 cells. BA was slightly more effective than AZA in the A375 cells under normoxic conditions, which is probably not related to the CA IX inhibitory effects of the compound (see later in the text and Table 1) which is a much weaker CA IX inhibitor compared to AZA. For HT29 cell line, AZA acted as a much more potent inhibitor of CA IX in the conditions of the experiment (Figure 4(B)).

**Table 1.** Inhibition data of human CA isoforms CA I, II, IX, and XII with boric acid and the standard sulphonamide inhibitor acetazolamide (AAZ) by a stopped-flow CO_2_ hydrase assay.

Compound	K_I_^a^				
	hCA I	hCA II	hCA IX	hCA XII	
Boric acid	29.9 mM	41.0 mM	2.8 mM	6.2 mM	
AAZ	250 nM	12 nM	25 nM	5.7 nM	

^a^Mean from three different assays, by a stopped-flow technique^13^ (errors were in the range of ± 5–10% of the reported values).

**Figure 5.**
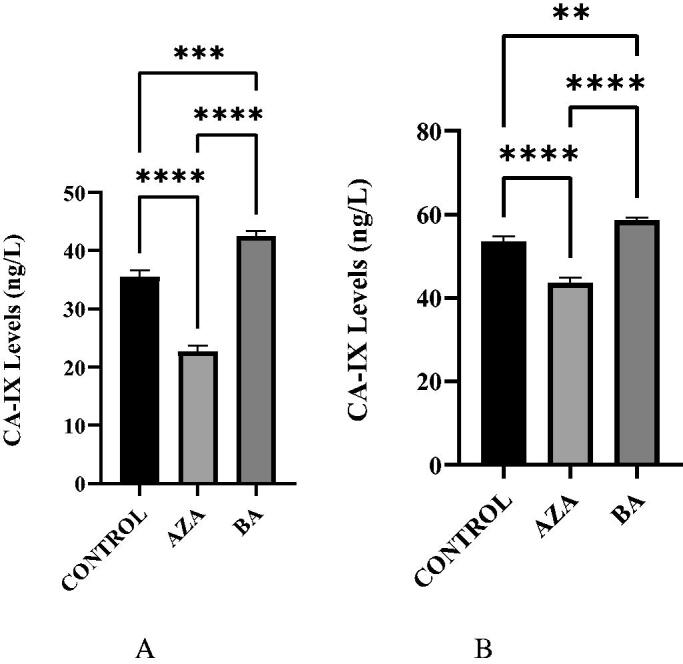
A. CA IX levels in AZA, BA, and control HT29 hypoxic cell cultures. CA IX levels showed a decrease in AZA-treated cells, however, an increase was seen in BA-treated cells (*p* < .0001 and *p* = .0004). B. CA IX levels in AZA, BA, and control HT29 normoxic cell cultures. CA IX levels were significantly reduced in AZA-treated cells but a slight increase is seen in BA-treated cells (*p* < .0001 and *p* = .0026).

In HT29 cells, both in normoxic (Figure 5(A)) and hypoxic (Figure 5(B)) conditions, the CA IX levels were decreased after treatment with AZA and were observed slightly increased after treatment with BA. This does not correlate with the *in vitro* inhibitory effects of these compounds against CA IX (Table 1), proving that some other mechanisms are responsible for the effect. We hypothesise that BA may interfere with the expression of the enzyme by a mechanism which is not elucidated at the moment.

As seen from data in Table 1, indeed BA (and many other inorganic small molecules, including inorganic anions)^9^ is a weak, millimolar inhibitor of four human (h) CA isoforms, hCA I, II, IX, and XII. hCA IX was the most effectively inhibited isoform, with a K_I_ of 2.8 mM, whereas the cytosolic hCA I and II were one order of magnitude less sensitive (with K_I_s of 29.9 − 41.0 mM). hCA XII was also inhibited in the low millimolar rage (Table 1). On the contrary, AZA is a nanomolar inhibitor of hCA II, IX, and XII; being less effective (micromolar range) against hCA I. Thus, the cell data reported here are probably due to a combination of effects, among which the CA IX inhibition is just one of them, with other phenomena probably occurring which are not elucidated at the moment.

## 4. Discussion and conclusion

Melanoma is a result of a malignant transformation of melanin-producing cells called melanocytes, though the treatment of melanoma has improved over the years with 1 out of 2 patients surviving 5 years after diagnosis when treated with combination therapy, additional approaches are needed for patients found to be resistant to currently available therapies^15^. The same can be said for colon cancer, although not considered as aggressive as melanoma, it is still one of the most common cancers seen in adults with approximately 800,000 deaths per year globally^16^. There are several reports supporting that boron compounds, including BA, show anticancer properties. One of the early such studies, using data from the NHANES III database reported that the risk of prostate cancer in US men is inversely proportional to the dietary intake of boron^17^. In another study, nude mice were treated with BA after being injected with androgen-sensitive LNCaP prostate cancer cells, which caused a decrease in tumour growth of 25 − 35%, along with a decrease in plasma PSA levels of 88%^18^. A similar decrease in cell proliferation was seen in DU-145 and PC-3 cell lines in a dose-dependent manner of BA^19^. BA was tested on other tumours, such as for example breast cancer^20^. Al-Ali et al tested BA on seven different cell lines and confirmed the compounds anti-carcinogenic properties^21^. In our study, we observed that BA is a weak inhibitor of CA IX, having also some effects on cancer cell proliferation in two cell lines. A375 and HT29 cell lines were treated with ascending concentrations of BA and the growth inhibition was determined in as dose-dependent manner. There are several other studies in the literature demonstrating the anti-carcinogenic effects of BA on HT29^21^^,^^22^ and SK-MEL 80^23^ cell lines.

We measured the CA IX levels in BA-treated cells and compared them with AZA-treated cells and control. We observed that A375 and HT29 cells treated with AZA under normoxic or hypoxic conditions had decreased CA IX levels (*p* < .0001). CA IX levels were also significantly reduced in BA treated A375 cells (*p* < .0001), however, the opposite effect was observed in BA treated HT29 cells (*p* < .05), through a mechanism which is not presently understood. AZA was effective in reducing CA IX levels in all cells, but we noticed that BA was more efficient than AZA in A375 cells, both in normoxic and hypoxic conditions, which does not correlate with the *in vitro* CA IX inhibitory effects of the two compounds. This effect is difficult to explain considering only the CA IX inhibitory effects and probably reflects the fact that BA may have many other targets in cancer cells, apart the enzymes considered here.

Our results prove that CA IX was inhibited by BA in A375 cell lines, probably due to BA-binding to the Zn(II) ion located at the active site of the enzyme. The increase of CA IX levels in HT29 cells shows a possibility of BA triggering an overexpression of the enzyme. Such phenomena deserve to be further investigated. In conclusion, BA inhibited cell proliferation of both A375 and HT29 cell lines in a dose-dependent manner. It caused a decrease in CA IX levels in A375 cells. In contrast, a slight increase in CA IX levels was seen in HT29 cells.
